# Chemistry and Neurotrophic Activities of (–)-Talaumidin and Its Derivatives

**DOI:** 10.3389/fchem.2020.00301

**Published:** 2020-04-23

**Authors:** Kenichi Harada, Miwa Kubo, Yoshiyasu Fukuyama

**Affiliations:** Faculty of Pharmaceutical Sciences, Tokushima Bunri University, Tokushima, Japan

**Keywords:** talaumidin, neurotrophic activities, PC12, regenerative activity, primary cultured rat cortical neurons

## Abstract

(–)-Talaumidin (**1**), a 2,5-biaryl-3,4-dimethyltetrahydrofuran lignan isolated from Aristolochia arcuata Masters, exhibits significant neurite-outgrowth promotion and neuroprotection in primary cultured rat cortical neurons and in NGF-differentiated PC12 cells. The first enantioselective total synthesis of **1** was achieved by a flexible and reliable synthetic pathway involving an Evans asymmetric aldol reaction, as well as a stereocontrolled hydroboration and Friedel–Crafts arylation, to construct the four contiguous chiral centers on the tetrahydrofuran (THF) ring of **1**. In order to investigate the stereochemistry–activity relationship of **1**, a systematic synthesis of all diastereomers of **1** was accomplished by applying the synthetic strategy used for natural product **1**. The evaluation of neurite-outgrowth promotion by all of the synthesized diastereomers indicated that the (–)-(1*S*,2*R*,3*S*,4*R*)-isomer **1e** was significantly more active than naturally occurring **1**. Additionally, we established a synthetic methodology for talaumidin derivatives that could be used to prepare a variety of analogs in a few steps and on a large scale. The synthesized racemic analog *rac*-**1e** (**56a**) exhibited neurite-outgrowth promoting activity in NGF-differentiated PC12 cells to the same degree as the optically active (–)-**1e**, revealing that a relative configuration bearing all-*cis*- substituents is important for potent neurotrophic activity, whilst the absolute configuration does not affect activity. Fourteen analogs based on (±)-**56a** were prepared via the same synthetic methodology. Among them, **56b** with a methylenedioxy group on both benzene rings was found to exhibit the most significant neurite outgrowth promotion. In addition, **56a** and **56b** induced regeneration of the mouse optic nerve *in vivo*, and their activity was higher than that of talaumidin, as well as their *in vitro* measured activity. Furthermore, the structure–activity relationship of **56b** indicated that the two benzene rings were essential structures, and that the methyl groups on the THF ring could enhance the neurotrophic activity. This result suggests that the two benzene rings of the talaumidin derivatives are essential structures for neurotrophic activity, while the two methyl groups on the THF ring can enhance neurite-outgrowth activity. Finally, it was observed that 1 and derivatives **56a** and **56b** exhibited potent regenerative activity in the injured mouse optic nerve *in vivo*.

## Introduction

Neurotrophins (NGF, BDNF, NT3, and NT4/5) are known to play essential roles in neuron survival, process outgrowth, and synaptic connectivity during development and nervous system plasticity in adults. Hence, they have a potential to become useful agents for neurodegeneration (Pardridge, 2002). Although, these polypeptide cannot cross the brain–blood barrier because of their high molecular weight and easily metabolize by peptidases under physiological conditions (Pardridge, 2002; Thoenen and Sendtner, 2002). Therefore, small molecules that can mimic the functions of neurotrophic factors might be promising alternatives for the treatment of neurodegenerative diseases (Xie and Longo, 2000; Massa et al., 2002). Neurotrophins also are able to promote process outgrowth and survival neuronal cells *in vitro*. Thus, we have been investigating neurotrophin-mimic small molecules from natural products based on rat cortical neuron cultures and PC12 cells, resulting in the discovery of interesting neurotrophic compounds (Huang et al., 2000, 2001; Fukuyama et al., 2002; Yokoyama et al., 2002; Kubo et al., 2009, 2010, 2012, 2013, 2015; Matsui et al., 2012).

**Figure 1:**
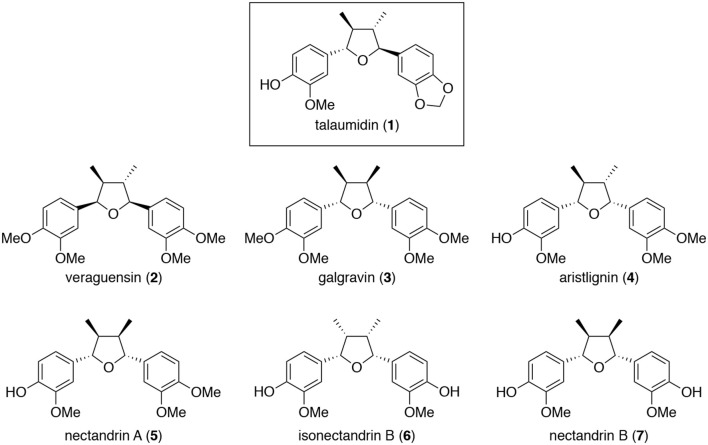
Structures of talaumidin (**1**) and analogs **2–7** (Zhai et al., 2005).

Talaumidin (**1**) is a 2,5-diaryl-3,4-dimethyltetrahydrofuran lignan (Figure 1), first isolated from the bark of *Talauma hodgsonii* Hook. f. and Thoms (Vieira et al., 1998). Talaumidin is categorized tetrahydrofuran lignans which are widely distributed in higher plants. Tertrahydrofuran lignans have attracted considerable attention due to their biological activities as cytotoxic activities (Vučković et al., 2007; Lin et al., 2010), DPPH-radical-scavenging activity (Mei et al., 2009), antioxidant activity (Piao et al., 2008), superoxide anion scavenging activities (Sasaki et al., 2013), growth and differentiation of osteoblastic MC3T3-E1 (Kiem et al., 2008), *anti*-HIV-1 activities (Zhang et al., 2007; Warashima et al., 2008), downregulate cyclooxygenase-2 (COX-2), inducible nitric oxide synthase (iNOS), and interleukin-1b (IL-1b) gene expressions in a dose-dependent manner in LPS-elicited mouse macrophages (Ma et al., 2007), inhibited NO production (Kim et al., 2014), *anti*-inflammatory activity (Wu et al., 2005), antimicrobial activities (Ding et al., 2014), antiproliferative activities against human cancer cell lines (Kim et al., 2011), and neurite-outgrowth promoting activity on PC12 cells (Kuroyanagi et al., 2008). On the other hands, biological activity of **1** has been documented as antiplasmodial activity (Abrantes et al., 2008) except for our reports, to date.

In our continuing studies on neurotrophic compounds, we isolated **1** from Blazilian plant *Aristolochia arcuata*. In addition to significant neurite-outgrowth promotion in primary cultured rat cortical neurons, we found that **1** and its analogs also exhibited neuroprotections against cell death induced by several insults (Zhai et al., 2004, 2005). Furthermore, **1**, belonging to a diaryltetrahydrofuran-type lignan, possesses a tetrahydrofuran ring bearing four contiguous stereogenic centers. These promising biological activities and the prospective selective preparation of the possible stereoisomers with regard to the four stereogenic centers of **1** make it an attractive synthetic target. In this review, we focus and summarize neurite-outgrowth promotion activities in primary cultured rat cortical neurons (Zhai et al., 2004) and in NGF-differentiated PC12 cells. Additionally, we describe the synthesis of **1** and all stereoisomers of **1** (Esumi et al., 2006; Fukuyama et al., 2008), and discuss structure–activity relationships between **1** and its analogs on PC12 cells (Harada et al., 2015). Furthermore, we report their regenerative activity toward mouse optic nerves as a neurotrophic activity *in vivo*, reinforcing their potential as therapeutic agents for neurodegenerative disease (Harada et al., 2018).

## Results and Discussion

Talaumidin (**1**) (Vieira et al., 1998) and its analogs, veraguensin (**2**) (Barata et al., 1978), galgravin (**3**) (Urzúa et al., 1987), aristlignin (**4**) (Urzúa et al., 1987), nectandrin A (**5**) (Le Quesne et al., 1980), isonectandrin B (**6**) (Le Quesne et al., 1980), and nectandrin B (**7**) (Le Quesne et al., 1980), were isolated from a methanol extract of the root of *A. arcuata* (*Aristolochiaceae*) by consecutive silica gel column chromatographies (Zhai et al., 2005).

### Neurite-Outgrowth Promoting Activity of Talaumidin in PC12 Cells

**Figure 2:**
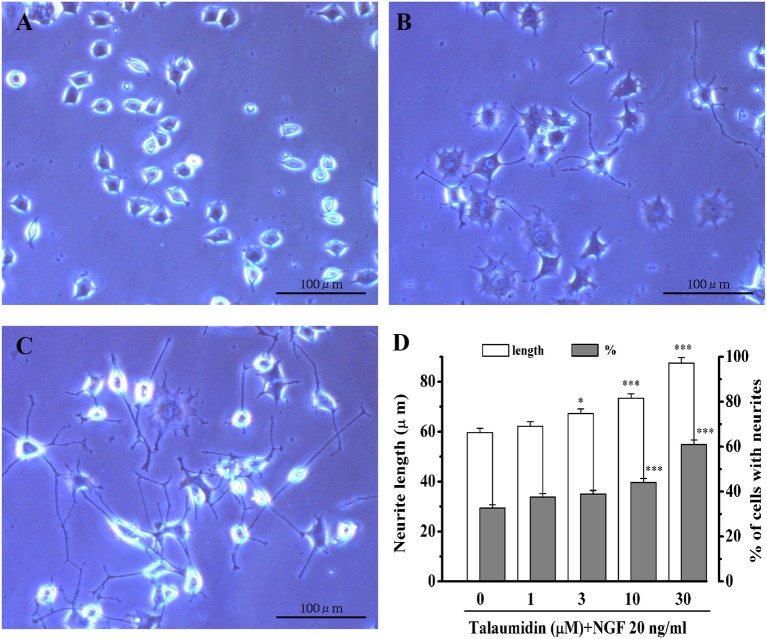
Neurite outgrowth promoting activities of **1** in NGF-differentiated PC12 cells. **(A)** Vehicle control (0.5% ethanol), **(B)** NGF 20 ng/mL, **(C)** NGF 20 ng/mL + talaumidin 30 μM, **(D)** quantitative analysis of neurite length and percent of cells bearing neurites. *P < 0.05, ***P < 0.001 vs. NGF alone (0 μM talaumidin).

Rat pheochromocytoma PC12 cells have been widely used as a model cells of neurons (Vaudry et al., 2002). When PC12 cells are stimulated with NGF, they cease growth and begin to grow neurites, eventually differentiating into a neuron-like phenotype. In the absence of NGF, talaumidin had no morphological effects on PC12 cells. In the presence of 20 ng/mL NGF, however, talaumidin promoted neurite outgrowth dose-dependently at concentrations of 1–30 μM, inducing longer average neurite length, as well as a higher percentage of neurite-bearing cells (Figure 2). These effects were validated through morphological observations (Figures 2C vs. **2B**) and quantitative analysis of neurites (Figure 2D).

### Neurotrophic Effects of Talaumidin in Primary Cultured Rat Cortical Neurons

**Figure 3:**
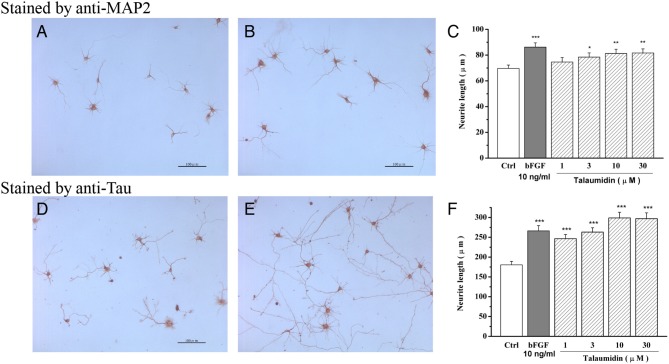
Neurite outgrowth-promoting activity of **1** shown by *anti*-MAP2 and *anti*-tau immunohistochemical staining in primary cultured rat cortical neurons. **(A,D)** Morphology of neurons in control groups, **(B,E)** Morphology of neurons in 10μM talaumidin groups, **(C,F)** quantitative analysis of dose-dependent manner. *P < 0.05, **P < 0.01, ***P < 0.001 compared with control (Ctrl) (Zhai et al., 2004).

In tissue sections and cell cultures, the *anti*-MAP2 antibody can stain neuronal cell bodies and dendrites but cannot use for efficient recognition of axons (Kaufmann et al., 1997). Alternatively, the *anti*-tau antibody reacts with tau proteins, which are distributed over the entire neuron surface, thus staining the cell body, dendrites as well as axons of neurons (Dotti et al., 1987). First, the morphological effects of talaumidin on cultured rat cortical neurons were evaluated by the *anti*-MAP2 staining method (Figures 3A,B). Talaumidin has been found to exhibit a significant promoting neurite outgrowth in the primary cultures of rat cortical neurons at concentration of 10 μmol/L. Measurements of each neuron stained by *anti*-MAP2 was performed by morphological analysis of process outgrowth brought on by talaumidin, and the quantitative results are shown in Figure 3C. It is obvious that talaumidin promotes process elongation dose-dependently at concentrations ranging from 3 to 30 μM. The longest processes stained with the *anti*-tau method are referred to as axon-like neurites, while others are referred to as dendrite-like neurites for clarity in the description of effects of **1** on neurite outgrowth. The morphological evaluation was carried out by *anti*-tau staining method (Figures 3D,E). According to expectations, **1** was observed to significantly promote dendrite-like processes, as well as axon-like processes at 10 μM. Quantitative analysis indicated that **1** enhanced process elongation in a dose-dependent manner at concentrations ranging from 1 to 30 μM (Figure 3F). Additionally, **1** also showed neuroprotective effects against serum deprivation-induced cell death in rat cortical neurons (Zhai et al., 2004).

### Evaluation of Neurite-Outgrowth Promoting Activity of Talaumidin and Other 2,5-diaryl-3,4-dimethyltetrahydrofuran Neolignans

**Figure 4:**
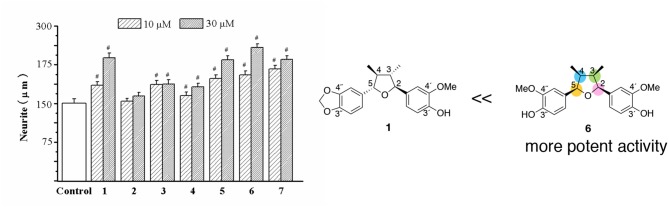
Comparison of the effects of compounds **1**–**7** on neurite outgrowth in primary cultured rat cortical neurons. ^#^*P* < 0.001 vs. control (Zhai et al., 2005).

The neurotrophic effects of 2,5-diaryl-3,4-dimethyltetrahydrofuran neolignans (**2**–**7**) were compared with talaumidin (**1**) (Zhai et al., 2005). Comparison of the effects of compounds **1**–**7** in the neurite-outgrowth assay indicated that **5**, **6**, and **7** were similar to **1**, but **2** and **4** had fewer effects than **1** (Figure 4). Especially, all-*cis*-substituted -type **6** showed the most potent neurite-outgrowth promoting activity at 30 μM. Furthermore, compounds **5** and **7**, whose stereochemistry are *trans*-*cis*-*trans* (4,5-*trans*, 3,4-*cis*, and 2,3-*trans*), presented similar activity of **1**. Curiously, *trans*-*cis*-*trans* **3** could not be attributed to effect. The above preliminary structure–activity analysis assumes that the stereochemistry of tetrahydrofuran ring and substituents on two benzene groups would make an important contribution to the enhancement of activity. It's very interesting challenge to investigate the relationship between stereochemistry and substituent and the neurotrophic activity of talaumidin.

### Total Syntheses of (–)-Talaumidin

Due to its unique structure and potent neurotrophic activities, talaumidin has been the target of extensive synthetic efforts over the years. To date, six syntheses have been reported. In 2006, we reported the first total synthesis of (2*S*,3*S*,4*S*,5*S*)-talaumidin *via* a flexible and stereo-controlled synthetic strategy (Esumi et al., 2006; Fukuyama et al., 2008). In addition, we determined the absolute configuration of (–)-talaumidin to be (2*S*,3*S*,4*S*,5*S*) during the course of the synthetic study. In 2007, Hanessian et al. attained the synthesis of unnatural (+)-talaumidin along with four tetrahydrofuran analogs (Hanessian and Reddy, 2007). In the same year, Kim et al. disclosed the stereoselective reductive deoxygenation/epimerization of cyclic hemiacetals and the synthesis of (+)-talaumidin (Kim et al., 2007). Alternative syntheses of talaumidin have additionally been reported by Matcha and Ghosh (2008), Rye and Barker (2009), and Xue et al. (2009). Synthetic studies of tetrahydrofuran-type lignans are actively continuing worldwide.

#### Synthesis of Talaumidin by Fukuyama et al.

**Scheme 1:**
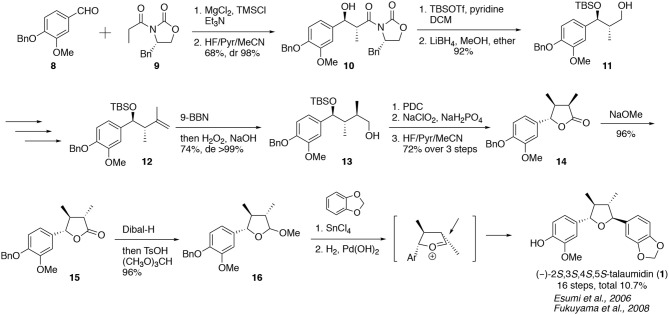
First synthesis of (–)-talaumidin by Fukuyama et al.

In 2006, we accomplished an enantioselective synthesis of (2*S*,3*S*,4*S*,5*S*)-talaumidin in advance of the other synthetic studies (Scheme 1) (Esumi et al., 2006; Fukuyama et al., 2008). The synthesis of **1** commenced with an *anti*-selective Evans asymmetric aldol reaction. The reaction of benzaldehyde **8** with chiral oxazolidionone **9** in the presence of MgCl_2_, gave rise to (2*S*,3*S*)-aldol adduct **10** in 98% de (Evans et al., 2002). Following the conversion of alcohol **11** to the exomethylene **12**, diastereoselective hydroboration of **12** was examined. Using 9-BBN, the reaction proceeded in >99% de, in accordance with the Cram rule (Houk et al., 1984). Although the generated chirality at C4 was opposite to the desired stereochemistry, the chiral center could be inverted to 4*S*-configuration by exposing lactone **14** to basic conditions. The last stage entailed a diastereoselective Friedel–Crafts arylation of cyclic acetal **16**, which afforded a single stereoisomer bearing the (2*S*,3*S*,4*S*,5*S*)-configuration. Finally, the total synthesis of (2*S*,3*S*,4*S*,5*S*)-**1** was completed by hydrogenolysis of the benzyl group. The first enantioselective synthesis of (2*S*,3*S*,4*S*,5*S*)-**1** was accomplished in 10.7% overall yield, over 16 steps. All spectroscopic data, such as NMR, HR-MS, IR, CD, [α]_D_ of synthesized (2*S*,3*S*,4*S*,5*S*)-talaumidin were identical to those of natural (–)-talaumidin. From these results, the absolute configuration of natural (–)-talaumidin was determined to be (2*S*,3*S*,4*S*,5S).

#### Synthesis of (+)-Talaumidin by Hanessian et al.

**Scheme 2:**
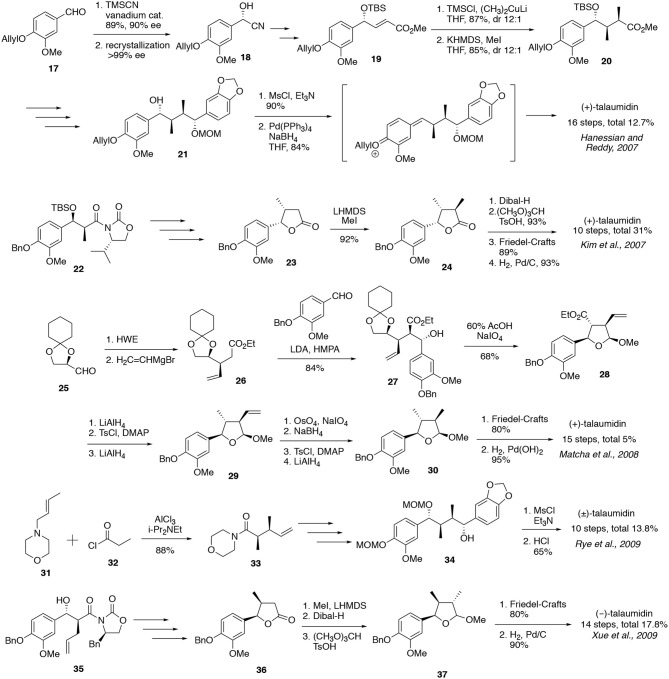
Total syntheses of talaumidin.

In 2007, Hanessian et al. reported a second synthesis of talaumidin, which was performed as part of a synthetic study on tetrahydrofuran-type lignan compounds (Scheme 2) (Hanessian and Reddy, 2007). The synthesis began with a chiral cyanohydrin **18**, in turn prepared by a catalytic asymmetric reaction of **17**, according to Belokon's protocol (Belokon et al., 2000). After Wittig olefination, 1,4-addition of **19** with dimethyllithium cuprate and TMSCl afforded *anti*-configuration in 12:1 dr. Subsequent α-alkylation of the ester with MeI *via* an enolate provided **20** with high diastereoselectivity. Then, a Grignard reaction with the aldehyde in the presence of CeCl_3_ provided **21** having the two (*R*)-hydroxy moieties. The key cycloetherification of **21** gave rise to the talaumidin skeleton in 90% yield through a quinone methide intermediate. Following deprotection, the total synthesis of (+)-talaumidin was attained in an overall yield of 12.7% over 16 steps.

#### Synthesis of (+)-Talaumidin by Kim et al.

Kim et al. investigated synthetic methodologies for the assembly of tetrahydrofuran lignans and the total synthesis of (+)-talaumidin was accomplished as a part of these studies (Kim et al., 2007). Talaumidin was constructed utilizing the Evans aldol, *anti*-selective dimethylation, and Friedel–Crafts reactions as key transformations. In addition, they established a synthetic strategy featuring Lewis acid promoted deoxygenation followed by epimerization of a hemiacetal, and accomplished the synthesis of five tetrahydrofuran-type lignans. The efficiency of Kim's synthesis was showed by the short step and high overall yield of 31%.

#### Synthesis of (+)-Talaumidin by Matcha et al.

In 2008, Matcha et al. synthesized (–)-talaumidin and (–)-virgatusin using the chiral pool approach in 15 steps with 5.0% overall yield. The synthesis of **1** commenced with the chiral starting material (*R*)-(+)-2,3-di-*O*-cyclohexylidine glyceraldehyde (**25**) which was derived from the chirality of D-mannitol (Chattopadhyay, 1996; Banerjee et al., 2005). The key step was a diastereoselective aldol reaction of the enolate derived from **26** with benzaldehyde **8**. The aldol **27** was obtained in 84% yield as a major product, accompanied by two diastereomers (dr 13:1.3:1). After several redox processes, a Friedel–Crafts arylation of acetal **30** followed by hydrogenolysis completed the synthesis of (–)-talaumidin. However, the indicated absolute configuration was not consistent with the optical rotation reported in other syntheses (Esumi et al., 2006; Hanessian and Reddy, 2007; Kim et al., 2007; Xue et al., 2009).

#### Synthesis of (±)-Talaumidin by Rye et al.

In 2009, Rye et al. reported a straightforward synthetic methodology for the preparation of tetra-substituted tetrahydrofuran lignans such as (±)-talaumidin and (±)-fragransin A_2_. The synthetic pathway began with an acyl-Claisen rearrangement to construct two successive tertiary stereocenters. After the introduction of an aryl group, the intramolecular cyclization of monoprotected 1,4-diol **34** gave the talaumidin skeleton. Removal of the MOM group completed the total synthesis of racemic talaumidin in an overall yield of 13.8%. Its analog, racemic franransin A_2_, was synthesized in the same manner, in an overall yield of 5.8%. Although it was a racemic synthesis, the economic synthesis of **1** was accomplished from inexpensive starting materials in 10 steps.

#### Synthesis of (–)-Talaumidin by Xue et al.

Xue et al. reported the total syntheses of (–)-talaumidin and (–)-galbergin. They combined Fukuyama's and Kim's strategies and applied the Evans aldol and Friedel–Crafts reactions to control the stereochemistry of successive four chiral centers. Overall yields of (–)-talaumidin and (–)-galbergin were 17.8 and 16.9%, respectively.

#### Stereoselective Construction of Tetrahydrofuran-Type Lignan Skeleton

In these syntheses, some common procedures were established in order to construct the four chiralities of talaumidin. Evans aldol reaction has been utilized by three research groups and proven to be an optimal procedure forming successive chiral centers at C2 and C3 of **1**. On the other hand, the third chiral center at C4 was constructed by α-substitution of carbonyl group, except for Fukuyama's synthesis. Finally, Friedel–Crafts reaction or intramolecular etherification have been adopted for the control of chirality at C5. The both reactions are found to be appropriate to control the 4,5-*trans*-configuration. These procedures would be a standard strategy for the synthesis of tetrahydrofuran-type lignans.

### The Relationship Between Stereochemistry and Neurite-Outgrowth Activity of Talaumidin (1)

**Scheme 3:**
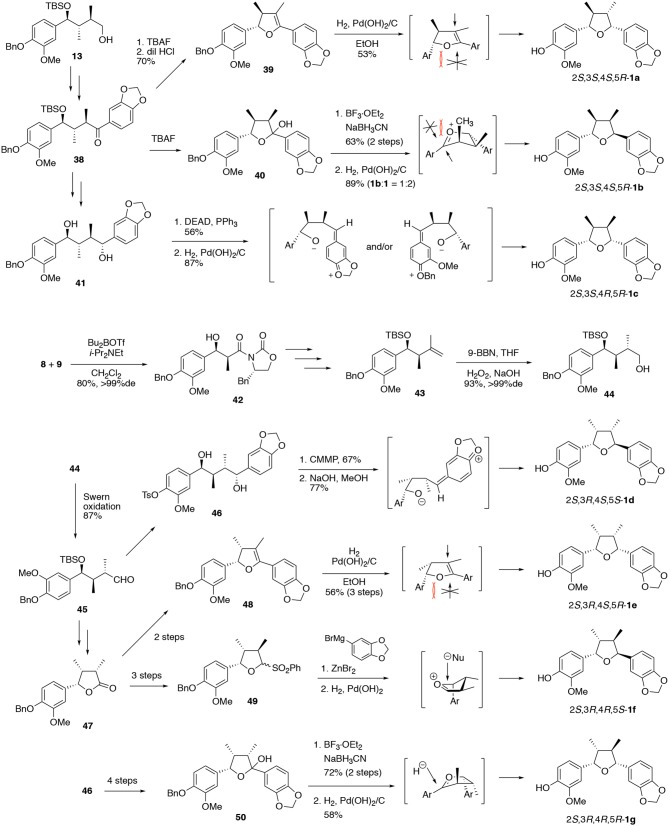
Systematic synthesis of seven diastereomers of (–)-talaumidin.

Following our asymmetric total synthesis of (–)-talaumidin, we embarked on structure–activity relationship (SAR) studies with the aim of potential drug discovery based on talaumidin. Initially, the relationship between stereochemistry and the neurotrophic activity of talaumidin was investigated. As **1** possesses four successive asymmetric carbons on the THF structure, seven diastereomers **1a–1g** are possible, in addition to the enantiomer of each. The successful control of the four contiguous stereocenters in an asymmetric synthesis of all seven diastereomers would be crucial for the elucidation of the relationship between stereochemistry and neurotrophic activity, and likewise be an important achievement from the viewpoint of organic synthetic chemistry. In 2015, we published the systematic synthesis of talaumidin diastereomers and their evaluation of neurotrophic activity (Scheme 3) (Harada et al., 2015).

Diastereomers **1a–1c** having (2*S*,3*S*)-configuration were synthesized from **13**, which was an intermediate in the synthesis of **1** (Esumi et al., 2006; Fukuyama et al., 2008). After the introduction of the aryl group, the resulting ketone **38** was converted to dihydrofuran **39**. The stereoselective hydrogenation of dihydrofuran **39** provided (2*S*,3*S*,4*S*,5*R*)-**1a** as a sole stereoisomer. Next, treatment of hemiacetal **40** with BF_3_·OEt_2_ followed by reduction with NaBH_3_CN furnished (2*S*,3*S*,4*R*,5*S*)-**1b**. In this reductive sequence, the hydride nucleophile approaches from the opposite face to the adjacent methyl group. On the other hand, the synthesis of (2*S*,3*S*,4*R*,5*R*)-**1c** was attained by applying a Mitsunobu-type reaction with 1,4-diol **41** using DEAD and PPh_3_. Noteworthy, the stereoselective cyclization proceeded with net retention of original configurations at C1 and C4. This surprising stereoselectivity was rationalized by preferential elimination of the PPh_3_-activated hydroxy group over the normal substitution pathway, giving rise to quinone methide intermediates (Harada et al., 2011b). The steric hindrance between a methyl group and the adjacent aryl group forces the product to adopt the desired conformations, resulting in the construction of (2*S*,3*S*,4*R*,5*R*)-configuration.

Subsequently, the synthesis of stereoisomers **1d–1g** with (2*S*,3*R*)-configuration was achieved *via* common intermediate **44**. The (2*S*,3*R*)-configuration of **44** was constructed by *syn*-selective Evans aldol reaction between **8** and **9** in 80% yield with >99% de. According to the same synthetic procedure for (–)-**1**, the key intermediate **44** was derived from **43** by hydroboration with 9-BBN. With **44** in hand, the synthesis of (2*S*,3*R*,4*S*,5*S*)-**1d** was firstly attained by a cyclization of diol **13** under conditions of Mitsunobu-type reaction (Harada et al., 2011a). In this case, the benzyl group was converted to a tosylate prior to cyclization, in order to enhance the selectivity eliminating the hydroxy group at C4. Next, hydrogenation of dihydrofuran **11b** gave all-c*is*-substituted (2*S*,3*R*,4*S*,5*R*)-**1e** with high diastereoselectivity. In addition, the C4 position of lactone **21** was epimerized, and then led to **23** by reduction of the lactone, followed by sulfonation. In accordance with Ley's method (Brown et al., 1989; Kim et al., 2007), a Grignard reaction with zinc bromide introduces the methylenedioxy benzene moiety from the β-face to avoid steric hindrance between the aryl groups (Harada et al., 2011a). Then, (2*S*,3*R*,4*R*,5*S*)-**1f** was synthesized by removal of the benzyl group. Finally (2*S*,3*R*,4*R*,5*R*)-**1g** was synthesized by the reduction of **27** with NaBH_3_CN/BF_3_·OEt_2_ conditions. Regarding this stereochemistry, the epimerization at C3 proceeded spontaneously in order to decrease the steric hindrance from the methyl group.

These synthetic studies provided useful information for the analysis of stereochemistries of tetrahydrofuran lignans. The characteristic ^1^H and ^13^C NMR data of **1** and **1a–1g** are summarized in Tables 1, 2. Although coupling constants are indecisive, chemical shifts play a role in identifying relative stereochemistries on the THF ring. In the case of 2,3-*trans*- and/or 4,5-*trans*-configurations, the signal of methyl group appears at 0.99–1.04 ppm. In contrast, 2,3-*cis*- and/or 4,5-*cis*-oriented methyl groups are shielded by the adjacent aromatic ring to result in upfield shift of the signal at 0.59–0.69 ppm. Moreover, the relative 2,3-stereochemistry is also able to be confirmed by ^13^C NMR. The chemical shifts of benzylic carbon are at 85.7–88.5 ppm for 2,3-*trans* and at 82.7–84.8 ppm for 2,3-*cis*, respectively. On the other hand, the relative configuration of 3,4-dimethyl groups can be distinguished by the chemical shifts of ^13^C NMR. The signal of 3,4-*trans*-dimethyl groups appears at 13.8–15.1 ppm, whereas 3,4-*cis*-dimethyl groups have lower chemical shifts around 9.4–12.9 ppm. These results of NMR experiments are consistent with those of natural products **2**–**7** and useful for the determination of relative stereochemistries of tetrahydrofuran type lignans.

**Table 1:** Characteristic ^1^H NMR data (δ_H_ (*J* in Hz)) for talaumidin (**1**) and its diastereomers **1a−1g**.

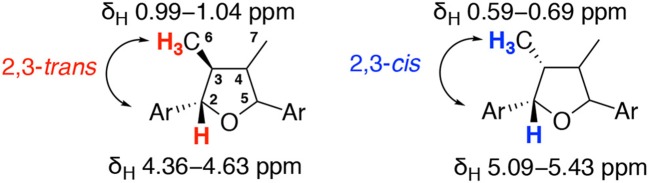								
**Positions**	**1**	**1a**	**1b**	**1c**	**1d**	**1e**	**1f**	**1g**
6 and 7	1.02 (5.8)	0.67 (7.0)	0.62 (7.3)	1.02 (6.6)	0.61 (7.1)	0.59 (6.1)	0.68 (6.5)	0.65 (6.8)
	1.02 (5.8)	1.04 (6.6)	0.99 (6.2)	1.02 (6.6)	1.00 (6.6)	0.61 (6.1)	0.69 (6.5)	1.04 (6.6)
3 and 4	1.76 (m)	1.75 (m)	2.43 (m)	2.28 (m)	2.42 (m)	2.65 (m)	2.25 (m)	1.74 (m)
	1.76 (m)	2.23 (m)	2.43 (m)	2.28 (m)	2.42 (m)	2.65 (m)	2.25 (m)	2.21 (m)
2 and 5	4.61 (9.1)	4.36 (9.3)	4.63 (9.3)	4.45 (6.4)	4.62 (9.3)	5.09 (6.4)	5.40 (6.0)	4.36 (9.3)
	4.61 (9.1)	5.09 (8.8)	5.43 (4.0)	4.46 (6.7)	5.43 (4.4)	5.09 (6.4)	5.40 (6.0)	5.10 (8.5)

**Table 2:** Characteristic ^13^C NMR data (δ_C_) for talaumidin (**1**) and its diastereomers **1a−1g**.

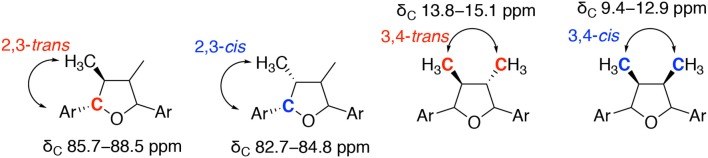								
**Positions**	**1**	**1a**	**1b**	**1c**	**1d**	**1e**	**1f**	**1g**
6 and 7	13.8	14.9	9.6	12.9	9.4	11.8	14.6	15.0
	13.8	15.1	11.9	12.9	11.8	11.8	14.7	15.1
3 and 4	50.9	46.0	43.5	44.5	43.5	41.5	43.8	45.9
	51.2	48.1	47.5	44.5	47.7	41.5	43.9	48.3
2 and 5	88.2	83.0	84.8	87.4	84.8	82.7	83.7	83.1
	88.5	87.5	85.8	87.5	85.7	82.8	83.7	87.4

**Figure 5:**
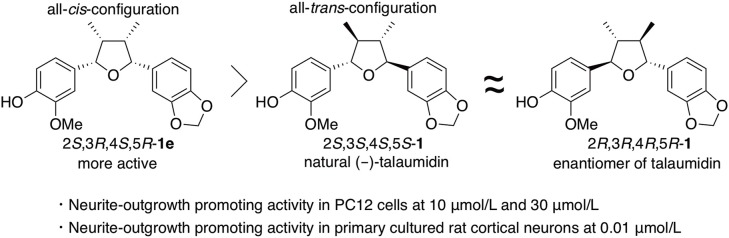
Relationship between stereochemistries and neurotrophic activity of talaumidin.

Once the synthesis of all diastereomers was complete, their neurotrophic activity was compared with that of natural talaumidin (Harada et al., 2015). Talaumidin (**1**) and isomers **1a**–**1g** were assessed their neurite-outgrowth promoting activity together with enantiomer of (–)-talaumidin. Consequently, the enantiomer of (–)-talaumidin exhibited activity similar to the natural product, and all synthesized compounds induced neurite-outgrowth promotion. Particularly, **1e** having all-*cis*-configuration was found to show more potent activity than naturally occurring talaumidin (Figure 5). Furthermore, their neurite-outgrowth promoting activity of stereoisomers in primary cultured rat cortical neurons was evaluated at 0.01 μM. The results indicated that all-*cis*-substituted **1e** also exhibited the most significant neurite-outgrowth promotion among all of the stereoisomers (Figure 5).

### Synthesis of Racemic Compound 56a and Relationship Between Substituents on the Benzene Ring and Neurotrophic Activity

**Scheme 4:**
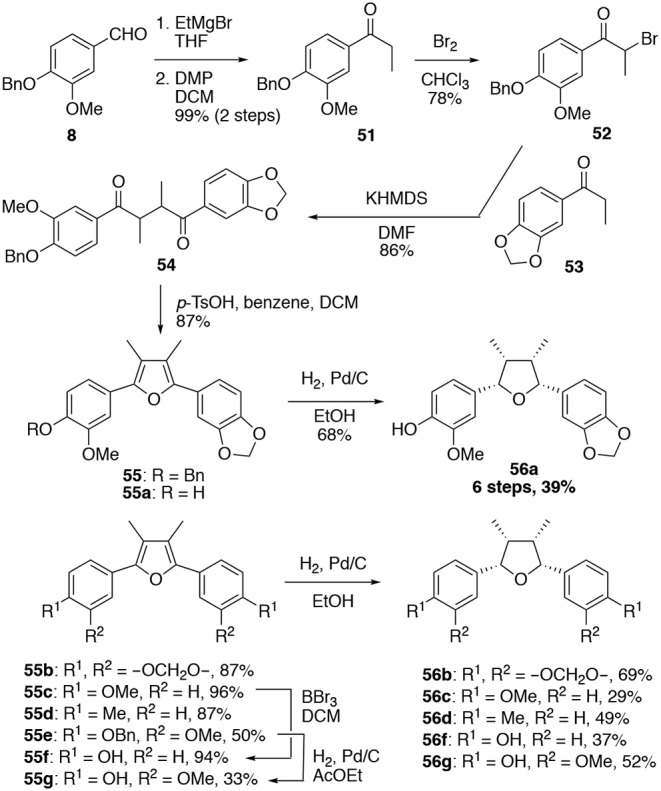
Step-economic synthesis of talaumidin derivatives.

(–)-Talaumidin and the stereoisomer (–)-**1e** were found to possess potent neurotrophic activity, however, preparative procedures of optically active (–)-talaumidin and (–)-**1e** required long synthetic steps and high cost. Therefore, drug discovery based on talaumidin necessitated a simplification of the structure and synthetic methodology for talaumidin derivatives. Then, we focused on the efficient synthetic methodology of talaumidin derivatives and exploration of new compounds that could be supplied on a large scale (Harada et al., 2018). In section The Relationship Between Stereochemistry and Neurite-Outgrowth Activity of Talaumidin (1), it was revealed that there are few difference in neurotrophic activity between both enantiomers of **1**. This result suggested that a racemic mixture of **1e** could have activity similar to optically active **1e**. Accordingly, racemic **1e** (**56a**) was decided on as the next target compound. The developed step-economic synthesis of *rac*-**1e** (**56a**) is shown in Scheme 4. The synthesis began with a Grignard reaction onto the commercially available benzaldehyde **8**, followed by a Dess–Martin oxidation. After bromination of **51**, the obtained bromide **52** was coupled with **53**, giving rise to a diketone **54** in 86% yield. Subsequently, Paal–Knorr furan synthesis of **54** under acidic conditions provided a furan compound **55** in good yield. At last, hydrogenation of the furan ring completed the synthesis of **56a** bearing all-*cis*-configuration. Consequently, the synthesis of racemic **1e** (**56a**) was accomplished in 6 steps with an overall yield of 39%. In addition, five novel talaumidin derivatives were prepared by applying this synthetic methodology.

**Figure 6:**
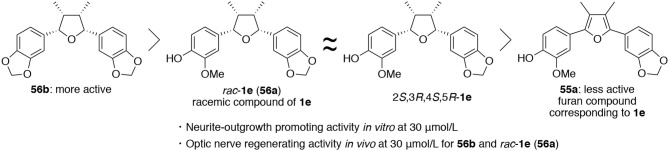
Relationship between substituents on the benzene ring and neurotrophic activity of talaumidin derivatives.

With six derivatives **56–ad, 56f**, and **56g** and their precursor furan compounds **55–ad, 55f**, and **55g** in hand, the neurite-outgrowth activity was evaluated in NGF-differentiated PC12 cells at 30 μM. As results, tetrahydrofuran compounds tended to promote neurite-outgrowth to a higher degree than furan-type compounds (Figure 6). Among the tetrahydrofuran compounds, **56b** having two methylenedioxyphenyl groups was found to exhibit the most significant activity. In addition, the step-economic synthesis of talaumidin derivatives allowed adequate quantities of samples to be prepared for *in vivo* experiments. Thus, we evaluated the optic nerve regenerating activity of talaumidin derivatives as an *in vivo* experiment. Remarkably, the all-*cis*-derivatives **56a** and **56b** showed high regenerative activity toward the injured optic nerve.

### The Role of Dimethyl and Diaryl Groups on THF Ring of Talaumidin Derivatives

**Figure 7:**
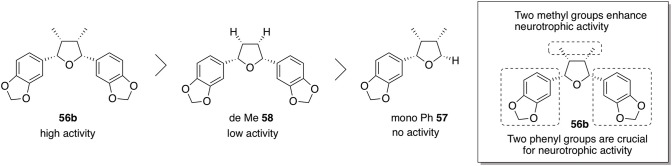
Investigation of SAR of talaumidin derivative **56b**.

Furthermore, the role of the phenyl and methyl groups on the THF ring of **56b** was examined as part of the SAR study. In order to determine which moieties were necessary for neurotrophic properties, **57** lacking one benzene ring and **58** lacking two methyl groups were prepared, and their neurotrophic activity was assessed in NGF-differentiated PC12 cells (Figure 7). It was found that monophenyl analog **57** exhibited no activity at all, whereas **58** had lower activity than **56b**. These results indicate that the two benzene rings of the talaumidin derivatives are essential structures for neurotrophic activity while the two methyl groups at C3 and C4 positions can increase the neurite-outgrowth activity.

### Mechanistic Study on Neurite-Outgrowth Activity of Staurosporine-Differentiated RGC-5 Cells

In 2018, Koriyama et al. examined the neurite-outgrowth promotion of talaumidin in the neuroretinal cell line, RGC-5 (Koriyama et al., 2018). They assessed the neurite outgrowth effect and elucidated a mechanism of its neurotrophic action. At concentrations ranging from 1 to 10 μM, talaumidin promoted neurite outgrowth dose-dependently in differentiated RGC-5 cells. Its neurite-outgrowth promoting activity was not altered by PD98059, an extracellular signal-regulated kinase inhibitor. On the other hand, LY29002, a PI3K inhibitor, decreased the talaumidin-mediated neurite outgrowth. These results indicate that the PI3K-Akt signaling is involved in downstream pathway in talaumidin-induced neurite-outgrowth activity of RGC-5 cells.

## Conclusion

In conclusion, the neurotrophic and protective activities of talaumidin have been found in several cellular models. Talaumidin can not only promote neurite outgrowth in NGF-differentiated PC12 cells but also enhance cell survival after NGF withdrawal in differentiated PC12 cells. These are coincident with the fact that talaumidin has neurotrophic effects on primary cultured rat cortical neurons. In addition, the neurotrophic activities of talaumidin are extended to neuroprotection, which are deleterious factors in Alzheimer's disease. Due to its interesting structure and neurotrophic activity, talaumidin have attracted considerable attentions from synthetic chemists. We achieved the first enantioselective synthesis of (–)-(2*S*,3*S*,4*S*,5*S*)-talaumidin using a Evans aldol reaction, hydroboration, and FriedelCrafts reaction. In addition, the systematic synthesis of all of the stereoisomers of (–)-talaumidin was accomplished, and their neurotrophic activity was evaluated. As results, the all-*cis*-substituted isomer **1e** showed more potent neurite-outgrowth promotion in NGF-differentiated PC12 cells than natural product talaumidin. Furthermore, we established a step-economic synthesis that could prepare a compound library based on talaumidin, and 14 derivatives were synthesized. As a result, compound **56b** having two methylenedioxyphenyl groups was found to show the most potent neurite-outgrowth promoting activity *in vitro*. Moreover, it was found that derivatives **56a** and **56b** could induce the regeneration of mouse optic nerve *in vivo*. These consequences indicate that talaumidin derivatives can be an innovative agent for neurodegenerative diseases such as glaucoma, depression, and Alzheimer's disease. Further mechanistic and pharmacological investigations of neurotrophic activities of talaumidin derivatives are currently ongoing.

## Author Contributions

KH, MK, and YF wrote the manuscript. All authors discussed the results and commented on the manuscript.

## Conflict of Interest

The authors declare that the research was conducted in the absence of any commercial or financial relationships that could be construed as a potential conflict of interest.
